# Migraine does not increase the risk of incident mild cognitive impairment five years later: Results of the population‐based Heinz Nixdorf Recall Study

**DOI:** 10.1002/alz70860_096563

**Published:** 2025-12-23

**Authors:** Martha Jokisch, Anna Lena Platzbecker, Janine Gronewold, Susanne Moebus, Andreas Stang, Börge Schmidt, Zaza Katsarava, Christian Weimar, Sara Schramm, the Heinz Nixdorf Recall Study Investigative Group

**Affiliations:** ^1^ Department of Neurology, University Hospital Essen, University of Duisburg‐Essen, Essen, Germany; ^2^ Institute of Urban Public Health, University Hospital of Essen, University of Duisburg‐Essen, Essen, Germany; ^3^ Institute of Medical Informatics, Biometry und Epidemiology, University Hospital of Essen, University of Duisburg‐Essen, Essen, Germany; ^4^ Department of Neurology, Christian Hospital Unna, Unna, Germany; ^5^ BDH clinic, Elzach, Germany; ^6^ Fliedner Fachhochschule Düsseldorf, University of Applied Sciences, Düsseldorf, Germany

## Abstract

**Background:**

During a migraine attack, patients with migraine experience non‐persistent cognitive impairment. However, some studies discuss migraine also as a risk factor for dementia in later life. The evidence, particularly with regard to mild cognitive impairment (MCI) as a precursor to dementia, is controversial. The aim of this study is to investigate the risk of incident MCI after five years in initially cognitively healthy participants with vs. without migraine.

**Method:**

2476 participants of the second (t1) and third (t2) examination of the prospective, population‐based Heinz Nixdorf Recall Study (t1: 2005‐2009, Ø62.9 years; t2: 2010‐2015, Ø68.1 years) with complete data on migraine status (t1), cognitive performance (T1&T2) and age‐appropriate cognition (t1) were included. MCI (at t2) was defined according to Winblad et al. (2004); migraine (at t1) was defined as self‐reported diagnosis. Log‐linear regression analyses with Poisson distribution were used to calculate the relative risk (RR) for incident MCI with 95% confidence interval (CI) for migraine vs. no migraine (unadjusted; adjusted for age, sex, education, alcohol consumption, smoking status, body mass index, depression according to directed acylic graph).

**Result:**

328 (13.2%) of the 2476 participants reported a migraine. Overall, 28 (8.5%) participants with migraine vs. 239 (11.1%) without migraine met the criteria for MCI at t2. There was no increased risk of incident MCI (unadjusted: RR 0.77, 95% CI 0.52‐1.14; adjusted: RR 0.77, 95% CI 0.52‐1.16) in participants with migraine.

**Conclusion:**

Even though migraine attacks impair cognitive performance, our data show no longitudinal association between migraine and MCI as a precursor to dementia. In further analyses, a distinction should be made between migraine with and without aura.

